# *p*-Curve and *p*-Hacking in Observational Research

**DOI:** 10.1371/journal.pone.0149144

**Published:** 2016-02-17

**Authors:** Stephan B. Bruns, John P. A. Ioannidis

**Affiliations:** 1 Meta-Research in Economics Group, University of Kassel, Kassel, Germany; 2 Departments of Medicine, Health Research and Policy, and Statistics, and Meta-Research Innovation Center at Stanford, Stanford University, Stanford, United States of America; Universiteit Gent, BELGIUM

## Abstract

The *p*-curve, the distribution of statistically significant *p*-values of published studies, has been used to make inferences on the proportion of true effects and on the presence of *p*-hacking in the published literature. We analyze the *p*-curve for observational research in the presence of *p*-hacking. We show by means of simulations that even with minimal omitted-variable bias (e.g., unaccounted confounding) *p*-curves based on true effects and *p*-curves based on null-effects with *p*-hacking cannot be reliably distinguished. We also demonstrate this problem using as practical example the evaluation of the effect of malaria prevalence on economic growth between 1960 and 1996. These findings call recent studies into question that use the *p*-curve to infer that most published research findings are based on true effects in the medical literature and in a wide range of disciplines. *p*-values in observational research may need to be empirically calibrated to be interpretable with respect to the commonly used significance threshold of 0.05. Violations of randomization in experimental studies may also result in situations where the use of *p*-curves is similarly unreliable.

## Introduction

The *p*-curve [1], the distribution of statistically significant *p*-values, has been used to infer that most studies actually analyze true relationships in the medical sciences [2] and in a wide range of disciplines [3] irrespective of whether these studies use experimental or observational research designs. However, other empirical surveys have documented an increased prevalence of *p*-values of 0.041–0.049 in the scientific literature over time [4,5], and the spurious excess of statistically significant findings in various types of both observational and experimental research [6,7] that have been attributed mostly to bias.

In this paper, we show that the *p*-curve cannot reliably distinguish true effects and null effects with *p*-hacking in observational research. Thus, using the *p*-curve to infer the presence of true effects or *p*-hacking in observational research is likely to result in false inferences. We use the term observational research to denote any study where there is no randomization in the comparison of the groups of interest. Observational studies comprise the large majority of the scientific literature with almost 300,000 observational studies compared to 20,000 randomized ones (experimental research) per year in PubMed [8].

Simonsohn et al. [1] who coined the distribution of statistically significant *p*-values “*p*-curve” have argued that true effects generate right-skewed *p*-curves whereas null effects should result in uniformly distributed *p*-curves. Jager and Leek [2] argue in a similar direction and describe the *p*-curve as a mixture of a uniform distribution and a beta distribution. The uniform distribution is supposed to describe those *p*-values generated by null effects and the beta distribution is supposed to capture *p*-values generated by true effects.

However, there is evidence that the incentive system of academic publishing fosters scientists to even engage in questionable research practices to search for and select statistically significant results [9,10]. We follow the notation of Simonsohn et al. [1] and denote “*p*-hacking” as the selection of statistically significant estimates for publication within each study. In experimental research, *p*-hacking includes choosing to report a subset of multiple dependent variables or adding observations until the effect of interest is significant [11]. Simonsohn et al. [1] argue that the *p*-curve is left-skewed in the presence of a null effect with *p*-hacking. The intuition is that *p*-hacked studies manipulate their estimates to achieve *p*-values that are just statistically significant but really small *p*-values close to zero are difficult to obtain by *p*-hacking. Hence, it is argued that true effects can be identified by right-skewed *p*-curves whereas left-skewed *p*-curves or a peak of *p*-values just below 0.05 indicate evidence for *p*-hacking.

*p*-hacking in observational research fundamentally differs from *p*-hacking in experimental research. A main decision in conducting regression analyses based on observational data is the selection of adjusting variables to be included in the regression so as to control for the impact of confounders. If an adjusting variable has an own effect on the dependent variable and is correlated with the variable of interest, excluding this adjusting variable from the regression induces omitted-variable bias (e.g. [12]). Omitted-variable bias represents a typical case of confounding that is not accounted for. For example, if exam grades are regressed on class attendance, it is likely to observe a positive and significant estimate if no adjusting variables are considered. However, pure class attendance may have in an extreme case actually no effect on the grades. But variables like the ability of the student or how hard the student has studied for the exam have an own effect on the grades and they are likely to be correlated with class attendance. If such variables are not considered as adjusting variables, the estimated effect of class attendance on grades is likely to be upwardly biased and may be statistically significant even if there is no true effect.

Even if the chosen regression specification exhibits only a tiny omitted-variable bias due to an incomplete set of adjusting variables, the *p*-value of the effect of interest can approach zero if the sample size is sufficiently large. This type of *p*-hacking generates right-skewed *p*-curves just as true effects do. Hence, if omitted-variable biases are used for *p*-hacking, the *p*-curve cannot distinguish between true effects and null effects with *p*-hacking in observational research.

We show by means of Monte Carlo simulations that null effects even with tiny omitted-variable biases in the range of *E*[*ρ*_*yx*_] = [0,0.01] (Where *ρ*_*yx*_ denotes the Pearson’s correlation coefficient between the dependent variable and the independent variable of interest) generate right-skewed *p*-curves. We further illustrate by using the effect of malaria prevalence on economic growth from 1960 to 1996 as an example how *p*-hacking results in right-skewed *p*-curves in observational research.

Our findings imply that inferences on true effects or *p*-hacking based on *p*-curves are likely to be flawed if observational research designs are considered. Furthermore, our findings provide further support to Schuemie et al. [13] that *p*-values in observational research may need empirical calibration to be interpreted.

## *p*-Hacking in Observational Research

In experimental research, *p*-hacking is explored as the search for statistically significant estimates by choosing dependent variables or covariates ex post, adding observations if the estimate is not significant, and reporting only subsets of experimental conditions [11]. John et al. [9] find in a survey that questionable research practices—that may be utilized to *p*-hack the estimate of interest—include among others the exclusion of data ex post, rounding down of *p*-values and framing an unexpected finding as having been predicted from the start.

These types of *p*-hacking are also likely to be observed in observational research. However, there is an additional major source of *p*-hacking in observational research. If the data is observational and regression coefficients are estimated to infer the relationship between two variables, many decisions have to be made, such as the choice of the functional form or the estimation technique. Most prominently, however, is the choice of the set of adjusting variables and this choice can strongly affect the estimate of the effect of interest. This flexibility results in a wide range of estimates from which statistically significant estimates can be easily selected. This type of *p*-hacking is sometimes known as multiple modelling [14], data snooping [15] or data-mining [16]. It may cause what is called a vibration of effects [17] and it is well known to be a key threat to the validity of inferences in observational research [14,18,19].

The estimate of interest primarily varies as omitted-variable biases are generated when the set of adjusting variables is varied. Omitted-variable biases differ substantially from biases that are discussed as *p*-hacking in experimental research. Omitted-variable biases lead to biased and inconsistent estimation of the effect of interest and they generate exactly the same statistical patterns as true effects do. Specifically, if the sample size increases, the *p*-value approaches zero irrespective of whether there is a true effect or a null effect with omitted-variable bias. This is different from *p*-hacking in experimental research in which the estimation of the effect of interest is unbiased and consistent due to randomization and *p*-hacking relies more on chance rather than on a systematic and asymptotical bias. Even a tiny omitted-variable bias can result in *p*-values that approach zero if the sample size is sufficiently large. Bruns [20] provides further discussion of *p*-hacking that is based on omitted-variable biases.

Simonsohn et al. [1] point out that the two determinants of the *p*-curve are the effect size and the sample size. This is true but the estimated effect size may be different from zero due to an omitted-variable bias rather than due to a true effect. This makes it impossible to use *p*-curves to distinguish between true effects and null effects.

We discuss *p*-hacking in observational research with a focus on omitted-variable biases as the variation of regression specifications is likely to be the major approach to select statistically significant estimates for publication. But other types of biases may also result in biased and inconsistent estimation of the effect of interest, e.g. simultaneity, misspecification of the functional form, and measurement error (e.g. [12]). Therefore, these types of biases may also result in right-skewed *p*-curves.

Though we discuss omitted-variable biases in the context of *p*-hacking, the scope of this bias is much larger. Schuemie et al. [13] show for the biomedical literature that even with best practice research designs the rate of false positives is vastly increased compared to what one might expect by chance if null effects are analyzed (5%). Best practice research designs denote here case-control, cohort, and self-controlled case series designs (e.g. case-crossover) in pharmacoepidemiology (drug safety) studies, but the concept can be extended to any other observational research field. This increased rate of false positives may be caused by omitted-variable biases that are not accounted for in the study design.

## *p*-Curve in Observational Research

We analyze the *p*-curve for observational research by using Monte Carlo simulations. We model *p*-hacking by generating random omitted-variable biases and by selecting the subset of statistically significant estimates from the set of generated estimates. We consider different strengths of *p*-hacking by using different sizes of omitted-variable biases and we consider a variety of sample sizes.

The data-generating process is given by:

$$y_{i}=\beta^{*}x_{i}+\gamma_{i}z_{i}+\epsilon_{i}$$ (1)

where the effect of interest is β*. We set β* = 0 to concentrate on the shape of *p*-curves in the presence of null effects with *p*-hacking. We use *i* = 1, …, 500000 iterations and in each iteration we draw *x*_*i*_, *z*_*i*_ and *ϵ*_*i*_ from a multivariate standard normal distribution and ensure exogeneity (*E*[*ϵ*_*i*_|*x*_*i*_, *z*_*i*_] = 0). The coefficient *γ*_*i*_ is different in each iteration and is used to generate omitted-variable biases as discussed below.

We generate these omitted-variable biases for the effect of interest (*β*) by estimating regressions that are based on the data generated in (1) but by omitting *z*_*i*_ from the regression:

$$y_{i}=\beta_{i}x_{i}+u_{i}.$$ (2)

The coefficient *β*_*i*_ of iteration *i* is potentially biased by omitted-variable bias. The expected size of this bias depends on the covariance between *x* and *z* as well as on *γ*_*i*_ (e.g. [12]) and is given by:

$$E\left[\beta_{i,ovb}\right]=\gamma_{i}\frac{Cov(x,z)}{Var(x)}.$$ (3)

As *x* stems from a multivariate standard normal distribution its variance is one and we set *Cov*(*x*, *z*) = 0.2 to concentrate on *γ*_*i*_ to model the omitted-variable biases. We draw *γ*_*i*_ in each iteration from an uniform distribution between 0 and *γ*^*max*^. The expected omitted-variable bias is then uniformly distributed and given by:

$$E\left[\beta_{i,ovb}\right]∼\text{unif}\left[0,E[\beta_{ovb}^{max}]\right]$$ (4)

where $E\left[\beta_{ovb}^{max}\right]=0.2\;*\;\gamma^{max}$.

We consider three different strengths of omitted-variable biases. Case 1 chooses $E\left[\beta_{ovb}^{max}\right]$ in a way that ensures a maximum expected Pearson’s correlation coefficient between *y* and *x* of $E\left[\rho_{yx}^{max}\right]=0.01$, Case 2 chooses $E\left[\beta_{ovb}^{max}\right]$ in a way that ensures $E\left[\rho_{yx}^{max}\right]=0.05$, and Case 3 chooses $E\left[\beta_{ovb}^{max}\right]$ in a way that ensures $E\left[\rho_{yx}^{max}\right]=0.1$ (S1 Appendix provides supplements to the simulation design). Note that the correlation between *x* and *y* is due to omitted-variable bias and not due to a true effect. According to Cohen [21] even a correlation of 0.1 is considered to be small. The maximum of our expected omitted-variable biases ranges from one tenth of a small effect to a small effect.

The sample size of each iteration *i* is drawn from a uniform distribution with a minimum of 50 and a maximum of *n*_*max*_ = 100,1000,10000,100000. These correspond to research done in different domains of observational research, ranging from relatively small studies (e.g. many studies on novel expensive biomarkers or uncommon conditions) to very large studies performed with large cohorts and big data.

Our modelling of *p*-hacking is conservative as we resample all variables in (1) in each iteration rather than resampling only *γ*_*i*_ until a statistically significant estimate is obtained. This ensures that there is no intensive search across different omitted-variable biases (by resampling only *γ*_*i*_) for a given dataset potentially implying that many estimates with extreme and unlikely biases would become statistically significant.

**Fig 1:**
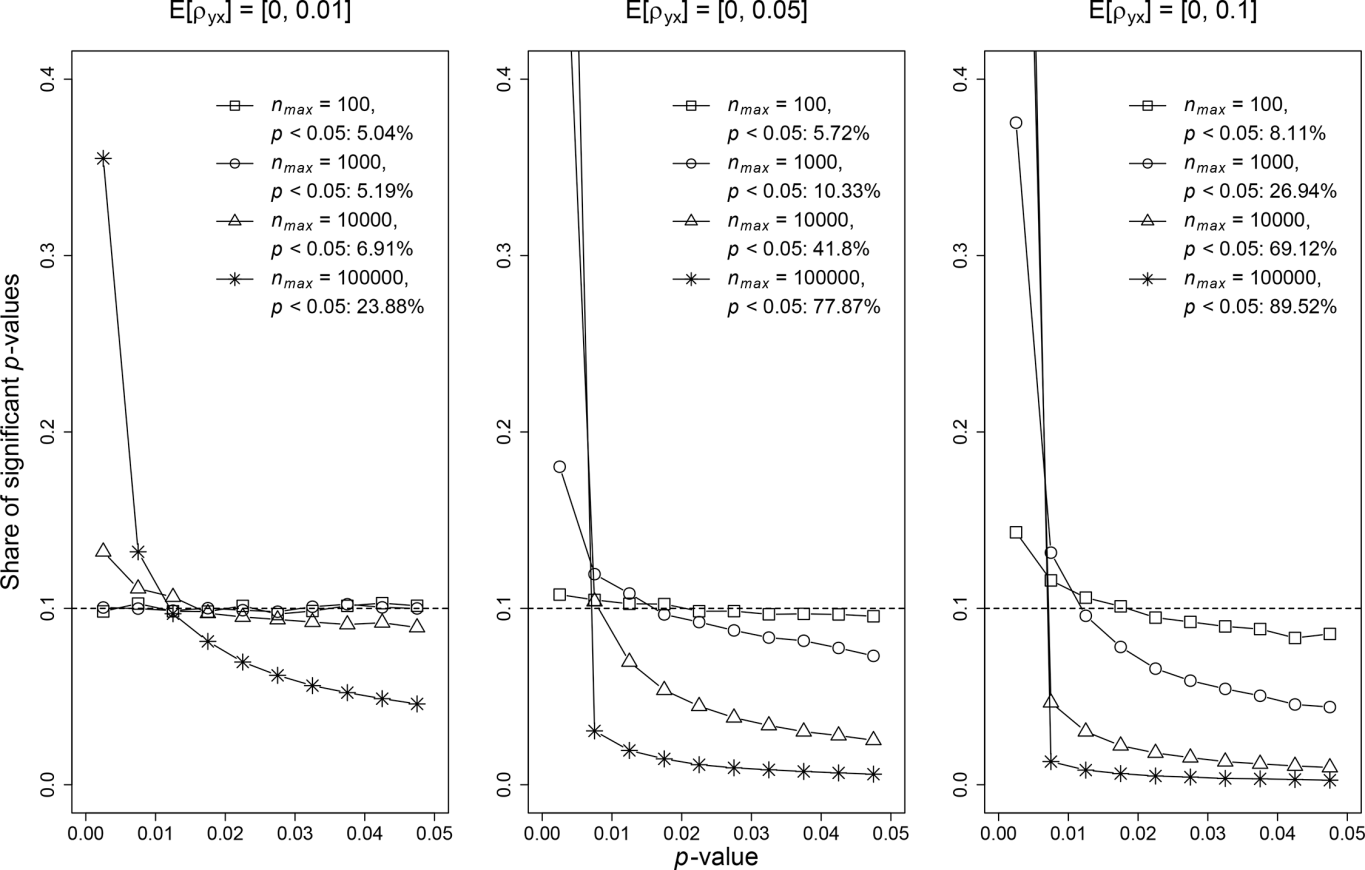
*p*-curves in the presence of *p*-hacking for different sample sizes. The y-axis depicts the share of statistically significant *p*-values. *n*_*max*_ denotes the maximum sample size drawn from a uniform distribution with a minimum of 50 and *p* < 0.05 denotes the share of statistically significant *p*-values from 500,000 iterations. The dashed line represents a hypothetical uniform distribution of *p*-values.

Simulation results are presented in Fig 1 and indicate that even for these relatively tiny biases the *p*-curves become right-skewed if the sample size is sufficiently large. Additionally, none of the *p*-curves is left-skewed or shows a peak just below the significance threshold of 0.05. According to Simonsohn et al. [1] a right-skewed *p*-curve indicates the presence of a true effect and left-skewed *p*-curves indicate *p*-hacking. Our results show that both can be false in observational research.

## Empirical Illustration

We use the effect of malaria prevalence on economic growth to illustrate that *p*-curves cannot always distinguish between true effects and null effects with *p*-hacking. The illustration is based on the literature that attempts to identify determinants of economic growth by using cross-country growth regressions (see [22] for an overview). We use the classic data set of Sala-i-Martin et al. [23] that is widely used in this literature. It contains as the dependent variable the annualized average growth rate of real GDP per capita between 1960 and 1996 and 68 variables that may potentially cause economic growth. For this illustration, we select 15 variables from these 68 variables that are likely to affect economic growth. Most of these variables are measured in 1960 or in the 1960s to avoid endogeneity due to simultaneity.

We focus here on the effect of malaria prevalence in 1966 on economic growth in the subsequent decades. Using Bayesian model averaging Sala-i-Martin et al. [23] show that the effect of malaria prevalence on economic growth is sensitive to model size with larger models rendering the variable insignificant. This indicates that malaria prevalence is likely to play a spurious role in smaller models due to omitted-variable biases that are resolved in larger models. Recent reviews of the literature do not consider malaria prevalence to be a genuine determinant of economic growth [24].

Based on these prior findings, it is safe to consider for the sake of illustration that an effect of malaria prevalence on economic growth does not exist. However, to make sure that the effect is exactly zero we create a new growth variable that differs from the real growth variable only with respect to malaria prevalence. To do this, we use the selected 15 variables that are likely to be relevant for economic growth. We generate the new growth variable by first estimating (S2 Appendix provides variable notation):

$$\begin{array}{l} GR6096=\alpha^{*}+\beta^{*}MALARIA+\delta_{1}OPEN+\;\delta_{2}FERTILITY\\ \;+\delta_{3}GDP60+\delta_{4}HIGHER.EDU+\delta_{5}INV.PRICE\\ \;+\delta_{6}LIFE.EXP+\delta_{7}PRIM.EDU+\delta_{8}POL.RIGHTS\\ \;+\delta_{9}POP+\delta_{10}TROPICA+\delta_{11}TRADE\\ \;+\delta_{12}BRIT.COL+\delta_{13}SPAIN.COL+\delta_{14}AREA.WATER\\ \;+\delta_{15}PUBLIC.INV+\epsilon \end{array}$$ (5)

and then using the original data and the estimates of α* and *δ*_1_, …, *δ*_15_ as well as the estimated residuals to generate a new growth variable *GR*6096_*new*_ by changing β* to zero (the actual estimate of β* in (5) is -0.00764 and clearly insignificant with a *p*-value of 0.224). This procedure allows us to sustain as much patterns of the real data as possible and simultaneously ensures that there is no effect of *MALARIA* on *GR*6069_*new*_. The correlation between the old and new growth variable is 0.987 (S2 Appendix provides supplements to the empirical illustration).

We analyze the *p*-curve of the effect of malaria prevalence on economic growth in the presence of *p*-hacking for a statistically significant effect that would demonstrate a detrimental impact of malaria prevalence on economic growth. The typical model size in the growth literature is characterized by seven independent variables [23]. Therefore, we consider regressions with malaria prevalence as the effect of interest and we include another 6 out of the 15 adjusting variables that were used to generate *GR*6096_*new*_. Selecting 6 out of 15 adjusting variables results in 5,005 different models that can be estimated:

$$GR6096_{new}=\alpha +\beta \;MALARIA+\gamma_{1}Z_{1}+\;\gamma_{2}Z_{2}+\gamma_{3}Z_{3}+\gamma_{4}Z_{4}+\gamma_{5}Z_{5}+\gamma_{6}Z_{6}+u$$ (6)

where *Z*_1_, …, *Z*_6_ denote the set of selected adjusting variables.

The data provides information on 99 countries for the selected variables. We draw random samples of countries with the sample sizes being drawn from a uniform distribution with a minimum of 50 and a maximum of 99. Using different samples of countries guarantees that the illustrative results are not specific to one sample and it mimics more realistically empirical literatures as the samples and sample sizes differ across studies.

**Fig 2:**
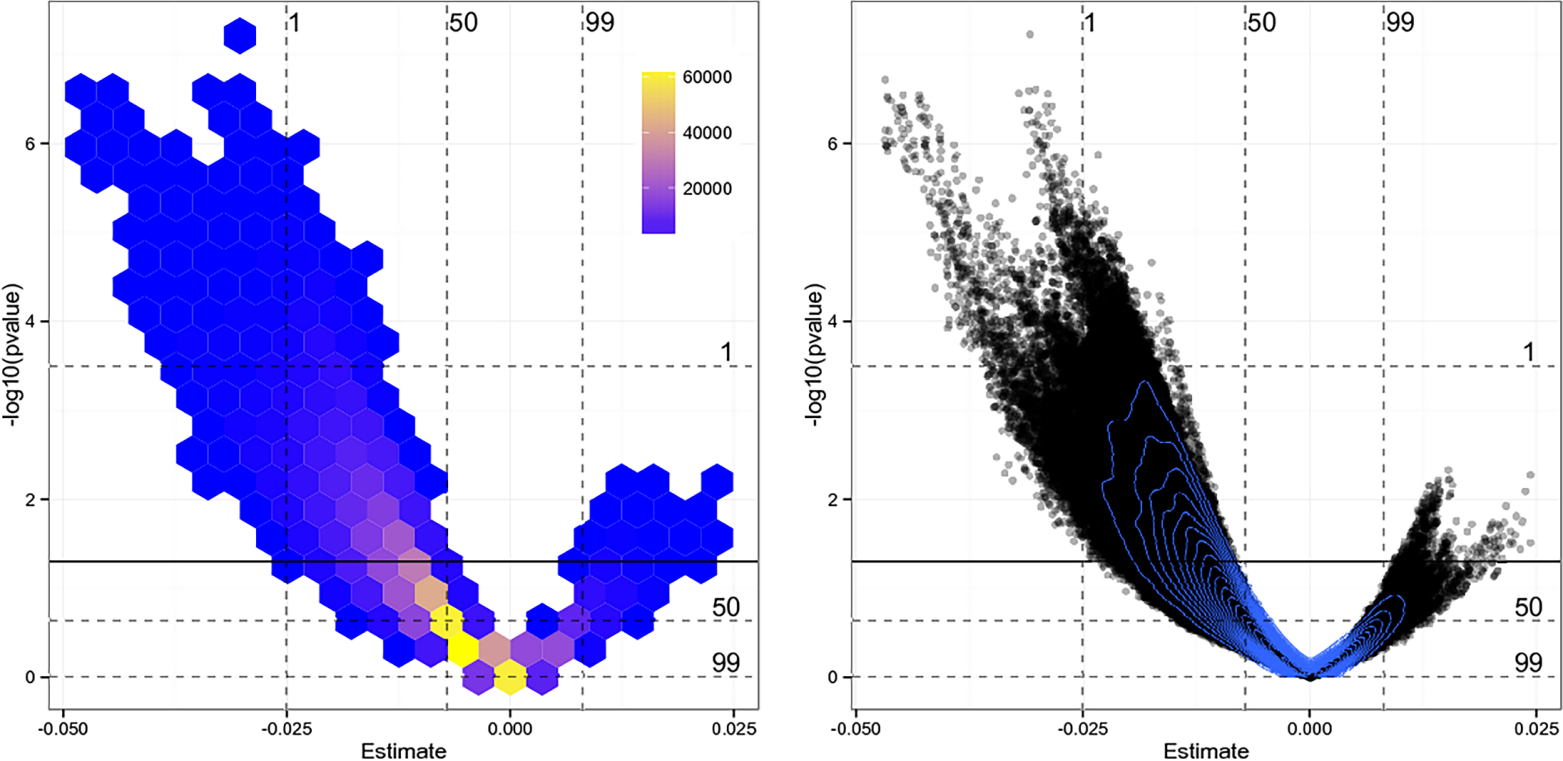
Vibration plot for the effect of malaria prevalence on economic growth. The vibration plot shows estimates of the effect of malaria prevalence in 1966 on the annualized average growth rate of real GDP per capita (1960–1996) on the x-axis. The y-axis shows transformed *p*-values of these estimates. The plot is based on 100 random samples of countries drawn from a uniform distribution with sample size between 50 and 99. For each sample of countries all 5,005 regression models are estimated resulting in 500,500 estimates of *β*. The dashed lines represent the 1, 50, and 99 quantiles of the distribution of transformed *p*-values and of the distribution of *β*, respectively. The solid line represents *p* = 0.05. Note that due to the transformation of *p*-values estimates above the line are statistically significant and below the line estimates are insignificant.

We illustrate the range of estimates of *β* that can be obtained by varying sets of adjusting variables and samples of countries by using a vibration plot [25]. For this purpose we use 100 random samples of countries and estimate for each sample the 5,005 models resulting in 500,500 estimates of *β*. The vibration plot illustrates that both positive and negative estimates of *β* are possible depending on the chosen adjusting variables (Fig 2). Negative estimates suggest that malaria prevalence has a detrimental impact on economic growth while positive estimates suggest that malaria prevalence enhances economic growth. Though effects with both signs are possible, most estimates result in a negative but statistically insignificant *β* (62.6%). Only 23.4% are negative and statistically significant at *p* < 0.05 (0.103% are positive and statistically significant and 13.9% are positive and insignificant at *p* < 0.05).

**Fig 3:**
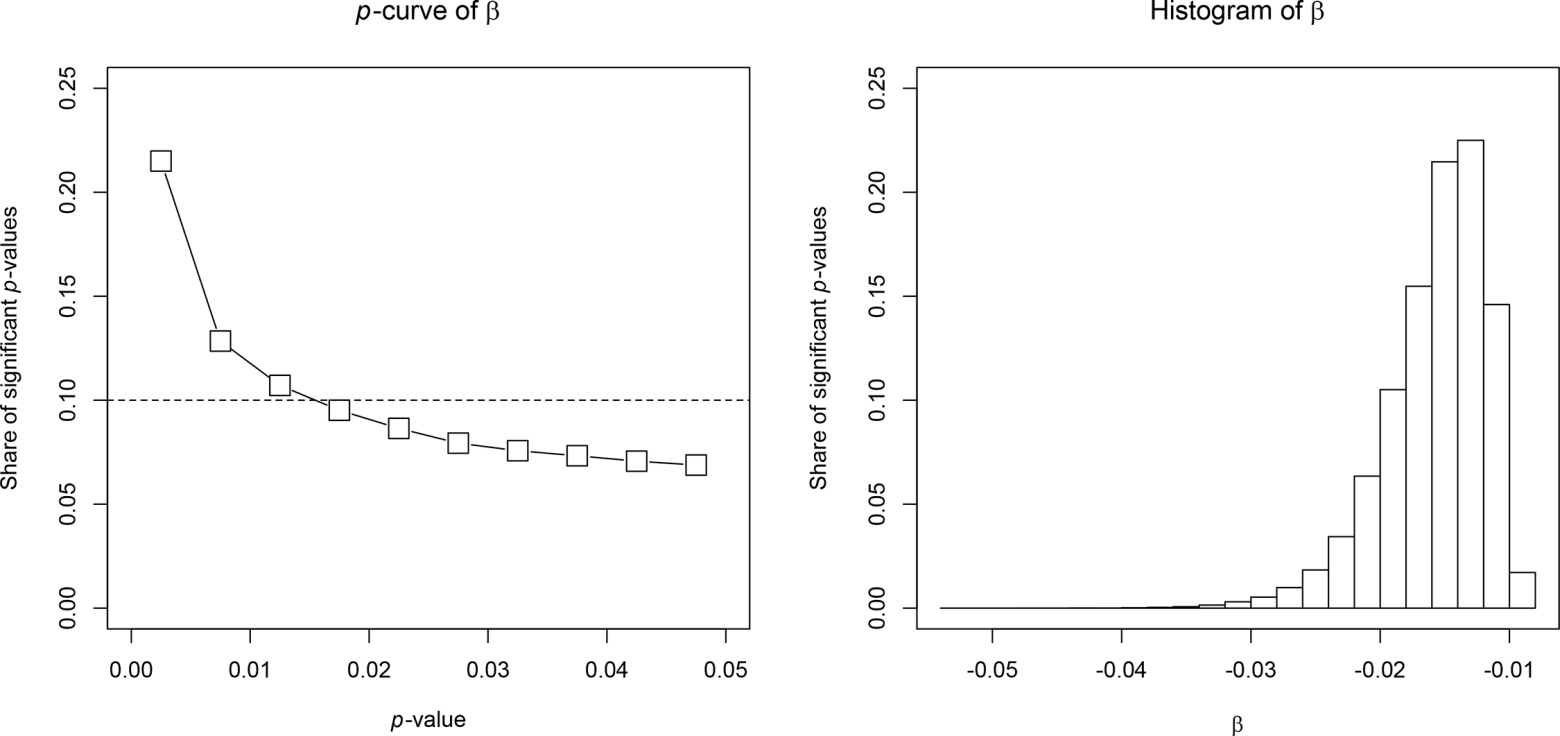
*p*-curve and histogram of estimates for the effect of malaria prevalence on economic growth. The *p*-curve of the estimated *β* of Eq (6) is shown in the left graph. The corresponding histogram of the estimated *β* is shown in the right graph. The y-axis displays the share of significant *p*-values. The graphs are based on the *p*-values of 100,000 statistically significant and negative estimates of *β*.

*p*-hacking is modelled by, first, drawing a random sample of countries with sample size between 50 and 99 and, second, browsing randomly through the 5,005 potential regression models. If a negative and statistically significant estimate of *β* is found, the estimate is selected and a new sample of countries is drawn and the search for a statistically significant and negative estimate of *β* starts again. If none of the 5,005 regression models results in a statistically significant and negative estimate of *β*, a new sample of countries is drawn and the specification search starts again. This illustrative example is less conservative and may be more realistic compared to the simulation design of the previous section as many regression specifications (potentially implying omitted-variable biases) are estimated for the same dataset and only if none of the regression specifications result in a negative and statistically significant estimate of *β*, a new sample of countries is selected. We do this until we obtain 100,000 statistically significant and negative estimates of *β*. Fig 3 shows the resulting *p*-curve and the selected estimates of *β*.

Consistent with our previous findings that are based on Monte Carlo simulations, the empirical illustration also reveals that *p*-curves become right-skewed in the presence of a null effect with *p*-hacking that is based on omitted-variable biases. There is also no sign of a peak of *p*-values just below the threshold of significance.

Following the recommendation of a reviewer, we also implemented this empirical illustration by using always the full sample of 99 countries to ensure that sampling errors do not confound our analysis. We do not expect sampling errors to cause right-skewed *p*-curves as sampling errors vanish with increasing sample sizes. This analysis also shows a right-skewed *p*-curve confirming that omitted-variable biases cause the *p*-curve to be right-skewed (S3 Appendix provides the results).

## Discussion

We show that *p*-hacking in observational research typically results in right-skewed *p*-curves that have been suggested to be evidence for a true effect [1,2]. The analyzed *p*-curves do not show any sign of being left-skewed though this was suggested as being evidence for *p*-hacking [1]. Our findings indicate that *p*-curves may neither identify true effects nor *p*-hacking in observational research.

Our findings are consistent with Schuemie et al. [13]. They show that if best practice designs are applied to observational data in biomedicine, the rate of false positives is vastly increased in the presence of a null effect. Schuemie et al. [26] further demonstrate that this increased rate of false positives is characterized by right-skewed *p*-curves. These findings suggest that even if best practice designs are applied to observational data, some biases remain that result in biased and inconsistent estimation of the effect of interest and *p*-values that approach zero with increasing sample sizes.

In this paper we demonstrate that one source for right-skewed *p*-curves in the presence of null effects is the omission of confounders resulting in omitted-variable biases. Other types of biases in observational research—such as misspecification of the functional form, simultaneity, and measurement errors—may also result in biased and inconsistent estimation of the effect of interest (e.g. [12]) and, thus, these types of biases may also result in right-skewed *p*-curves. The omission of confounders that is discussed here is likely to be a major source that biases the effect of interest asymptotically. However, the omission of confounders is not necessarily the result of *p*-hacking but may also stem from the lack of data availability, or lack of good knowledge about what confounders to adjust for. Even when observational research is done by seasoned experts, there is usually no consensus on what variables should be adjusted for. An empirical assessment of all 60 studies on pterygium risk factor epidemiology showed that there were no two studies that adjusted their models for the same variables [27].

The large potential impact of omitted-variable bias suggests that observational research might benefit from careful pre-specification of the analytical plan, when this research represents hypothesis testing. A large empirical survey of observational study protocols shows that even when these protocols are registered, their statistical analysis plans are almost never pre-specified [28], so there is plenty of room for improvement in this. For the large number of studies that are hypothesis generating and do not have pre-specified plans, their exploratory character should be transparently reported; different results obtained with different models should be acknowledged; and results for the model selected to be highlighted should be interpreted with great caution.

As we show, the extent of the right-skewed distortion of the *p*-curve with null effects is proportional to the amount of omitted-variable bias. When research is done with small sample sizes, small biases (reflected by $\left[\rho_{yx}^{max}\right]=0.1$) suffice to create major distortion. When large samples are considered, as typically seen in large cohorts or big data endeavors, even extremely tiny omitted-variable biases (e.g. $E\left[\rho_{yx}^{max}\right]=0.01$) will distort the *p*-values beyond repair. This may explain the extremely high failure of major inferences from observational studies to replicate in randomized trials [14,29]. It also should give us pause as to what extent observational big data can be trusted, when it is practically impossible to exclude the presence of such tiny biases that can totally invalidate the results [30].

One possibility is to use empirical calibration of *p*-values. Schuemie et al. [13] demonstrate for the biomedical literature that at least 54% of findings that claim statistical significance at 0.05 are statistically insignificant if empirically calibrated *p*-value are used. They calibrate the *p*-values by estimating the effects of drugs on the outcome of interest but where the drugs are not believed to cause the outcome. In these cases the null hypothesis of no effect should be true and an empirical null distribution can be derived that can be used to calibrate the *p*-values. These findings indicate that future research that uses large observational datasets should avoid evaluating *p*-values with respect to theoretical null distributions and the traditional threshold of 0.05. However, even empirical calibration is not always possible. A sufficient sample of non-contestable true positives and true negatives may not be available.

If *p*-hacking by means of omitted-variable biases is used to exaggerate true effects rather than rendering null effects statistically significant, the *p*-curve becomes right-skewed correctly indicating the presence of a true effect. Given the focus on statistical significance such an exaggeration of true effects may often occur when power is low. With regards to inferences on true effects by using *p*-curves, uncertainty remains whether a right-skewed *p*-curve indicates a null effect with *p*-hacking, a true effect, or a true effect with *p*-hacking to exaggerate the size of the effect.

Furthermore, we extend previous work by identifying some limits of using *p*-curves. Simonsohn et al. [1] introduced the *p*-curve primarily for experimental research and right-skewed *p*-curves may be a sign of true effects for these research designs. They show by means of simulation that for a specific type of *p*-hacking the *p*-curve becomes left-skewed. However, for other types of *p*-hacking there is no sign of left-skewed *p*-curves even in experimental research [31,32].

Moreover, we should mention that the small values of $E\left[\rho_{yx}^{max}\right]$ that we simulated for observational research may also occur in experimental research. Experimental research relies on randomization that ensures unbiased and consistent estimation of the effect of interest. But there is extensive literature that shows that a large proportion of seemingly experimental, randomized studies in fact are not properly randomized, or have many other biases that subvert randomization with substantial impact on their results [33]. Questionable research practices [9] can transform randomized trials to an equivalent of non-randomized observational studies and then the same issues surrounding *p*-hacking may apply [34].

Imbalances between the compared groups are possible in experimental studies. When they occur it is difficult to tell whether they represent chance or a sign of subverted randomization, i.e. that the trial is not really properly randomized and bias has interfered in the construction of the compared groups due to various reasons (e.g. a deficiency in proper allocation concealment). Moreover, results may differ with models using different adjustments even in randomized trials, particularly if randomization is not proper and thus a study is really observational, even if considered to be experimental/randomized. There is empirical evidence that different adjusted and unadjusted models in randomized trials may reach different conclusions [35]. In an empirical study of randomized trials published in the best clinical journals, the analysis of the primary outcome (adjusted or unadjusted) was different in the protocol versus the published papers and whenever only one of multiple analyses gave statistically significant results, this was almost always the analysis preferred by the authors [35].

The *p*-curve was also used to infer that most studies actually analyze true effects challenging previous claims [36]. Head et al. [3] find for text-mined *p*-values stemming from both experimental and observational research designs that for many disciplines the *p*-curves are right-skewed with some having a peak of *p*-values just below 0.05. Their main result is that even though *p*-hacking is ubiquitous it is of minor relevance as most studies in various disciplines analyze true effects. These results rest on the assumption that right-skewed *p*-curves indicate the presence of true effects, but this assumption may be false if observational research is considered. Even if right-skewed *p*-curves indicate true effects in experimental research, the presence of a right-skewed *p*-curve only implies that some of the studies analyze true effects [1]. Bishop and Thompson [31] illustrate that right-skewed *p*-curves may occur if only 25% of the considered studies analyze true effects.

Similarly, Jager and Leek [2] attempt to estimate the rate of false discoveries by assuming that the *p*-curve is a mixture of a uniform distribution and a beta distribution. The uniform distribution is supposed to represent *p*-values that stem from null effects whereas the beta-distribution is supposed to represent *p*-values that stem from true effects. Their main finding is that the false discovery rate in the medical literature is 14%. This means that the *p*-curve of the medical literature is best fitted by using 86% of a beta distribution and 14% of a uniform distribution. However, the analysis rests on the assumption that the right-skeweness of the beta distribution is due to *p*-values that stem from true effects. We show that this right-skeweness can easily be generated by null effects with *p*-hacking in observational research. The false discovery rate for this distribution could be anything, even 100%. Further problems in the analysis have been also discussed [37,38].

Little can be learned from such studies apart from an indication that *p*-curves may be right-skewed across some disciplines. The sources of this skewness, however, remain unexplained and uncertain. A much more promising empirical approach to the false discovery rate are replication studies as recently conducted by the Open Science Collaboration [39] and the Many Labs Replication Project [40].

## Supporting Information

S1 Appendix Supplements to the Simulation Design. (DOCX)

S2 Appendix Supplements to the Empirical Illustration. (DOCX)

S3 Appendix Empirical Illustration for the Full Sample of 99 Countries. (DOCX)

S1 Dataset Data of Simulation. (ZIP)

S2 Dataset Data of Empirical Illustration. (ZIP)
