# Supplementary material for: p-Curve and p-Hacking in Observational Research
Source: PLoS One. 2016 Feb 17;11(2):e0149144. doi: 10.1371/journal.pone.0149144 (PMC4757561; doi:10.1371/journal.pone.0149144)
Supplement: S1 Appendix — (DOCX) [file pone.0149144.s001.docx]

**S1 Appendix. Supplements to the Simulation Design**

The expected estimate of the regression coefficient of $x$ is given by (e.g. [1]):

$E\left[ \beta\right]=\beta^{*}+\gamma\frac{Cov\left( x,z \right)}{Var\left( x \right)}.$ (A1)

In order to obtain the correlation coefficient between $x$ and $y$ the regression coefficient has to be multiplied by ${sd(x)}/{sd(y)}$ in a bivariate regression. Given that $\beta^{*}=0$, the expected correlation coefficient is given by:

$E\left[ \rho_{xy} \right]=\gamma\frac{Cov(x,z)}{sd\left( x \right) sd(y)}.$ (A2)

We set $Cov\left( x,z \right)=0.2$ and $sd\left( x \right)=1$. $sd(y)$ is given by $\left( \gamma^{2}+1 \right)^{0.5}$ as $Var\left( y \right)=Var\left( \gamma z+\epsilon\right)=\gamma^{2}Var\left( z \right)+Var\left( \epsilon\right)$ and $Var\left( z \right)=1$ and $Var\left( \epsilon\right)=1$. For our simulation design follows that for a given $E\left[ \rho_{xy} \right]$ with $E\left[ \rho_{xy} \right]^{2}<0.04$, $\gamma$ is given by:

$\gamma=\sqrt{\frac{E\left[ \rho_{yx} \right]^{2}}{0.04- E\left[ \rho_{yx} \right]^{2}}}.$ (A3)

We consider three different cases with$E\left[ \rho_{yx}^{max} \right]=0.01$ $\left( \gamma^{max}\approx0.05 \right)$, $\left[ \rho_{yx}^{max} \right]=0.01$ $\left( \gamma^{max}\approx0.26 \right)$, and $E\left[ \rho_{yx}^{max} \right]=0.01$ $\left( \gamma^{max}\approx0.58 \right)$.

**References**

1. Greene WH. Econometric Analysis. 7th ed. Upper Saddle River, NJ: Prentice Hall; 2012.
