# Supplementary material for: p-Curve and p-Hacking in Observational Research
Source: PLoS One. 2016 Feb 17;11(2):e0149144. doi: 10.1371/journal.pone.0149144 (PMC4757561; doi:10.1371/journal.pone.0149144)
Supplement: S2 Appendix — (DOCX) [file pone.0149144.s002.docx]

**S2 Appendix. Supplements to the Empirical Illustration**

We use a subset of the data of Sala-i-Martin et al. [1] for the empirical illustration. Table 1 provides an overview of the variables that we use. The variable description is taken from Table 1 in Sala-i-Martin et al. [1].

Table 1: Description of the variables

| Variable Name | Description |
| --- | --- |
| $GR6096$ | Growth of GDP per capita at purchasing power parities between 1960 and 1996. |
| $GR{6096}_{new}$ | Same as $GR6096$ but with an effect of $MALARIA$ that is exactly zero. |
| $MALARIA$ | Index of malaria prevalence in 1966. |
| $OPEN$ | Number of years economy has been open between 1950 and 1994. |
| $FERTILITY$ | Fertility in 1960’s. |
| $GDP60$ | Logarithm of GDP per capita in 1960. |
| $HIGHER.EDU$ | Enrollment rates in higher education. |
| $INV.PRICE$ | Average investment price level between 1960 and 1964 on  purchasing power parity basis. |
| $LIFE.EXP$ | Life expectancy in 1960. |
| $PRIM.EDU$ | Enrollment rate in primary education in 1960. |
| $POL.RIGHTS$ | Political rights index. |
| $POP$ | Population in 1960. |
| $TROPICA$ | Proportion of country’s land area within geographical tropics. |
| $TRADE$ | Ratio of exports plus imports to GDP, averaged over 1965 to  1974. |
| $BRIT.COL$ | Dummy for former British colony after 1776. |
| $SPAIN.COL$ | Dummy variable for former Spanish colonies. |
| $AREA.WATER$ | Proportion of country’s land area within 100 km of ocean or  ocean-navigable river. |
| $PUBLIC.INV$ | Average share of expenditures on public investment as fraction of  GDP between 1960 and 1965. |

We estimate regression (5) in the article to obtain estimates of how the variables affect economic growth. Table 2 provides the corresponding regression output.

Table 2: Output of regression (5) in the article

|  | Estimate | Std. Error | *t*-value | *p*-value |  |
| --- | --- | --- | --- | --- | --- |
| (Intercept) | 1.94E-02 | 3.37E-02 | 0.574 | 0.567345 |  |
| MALARIA | -7.64E-03 | 6.23E-03 | -1.226 | 0.22388 |  |
| OPEN | 1.83E-02 | 5.85E-03 | 3.127 | 0.002447 | ** |
| FERTILITY | 1.17E-02 | 7.70E-03 | 1.513 | 0.134033 |  |
| GDP60 | -1.00E-02 | 2.81E-03 | -3.557 | 0.000627 | *** |
| HIGHER.EDU | -3.64E-02 | 4.45E-02 | -0.818 | 0.415702 |  |
| INV.PRICE | -6.97E-05 | 2.59E-05 | -2.694 | 0.008563 | ** |
| LIFE.EXP | 8.18E-04 | 3.58E-04 | 2.285 | 0.024866 | * |
| PRIM.EDU | 1.30E-02 | 8.06E-03 | 1.61 | 0.111221 |  |
| POL.RIGHTS | 3.75E-04 | 1.18E-03 | 0.317 | 0.752398 |  |
| POP | 6.18E-08 | 3.07E-08 | 2.017 | 0.046985 | * |
| TROPICA | -7.30E-03 | 5.34E-03 | -1.367 | 0.175311 |  |
| TRADE | 1.13E-02 | 5.32E-03 | 2.116 | 0.037383 | * |
| BRIT.COL | -3.83E-03 | 3.37E-03 | -1.137 | 0.258884 |  |
| SPAIN.COL | -1.23E-02 | 5.44E-03 | -2.265 | 0.026133 | * |
| AREA.WATER | 4.53E-03 | 4.23E-03 | 1.071 | 0.287144 |  |
| PUBLIC.INV | 9.96E-05 | 1.03E-04 | 0.967 | 0.336398 |  |
| \| Signif. codes: 0 ‘***’ 0.001 ‘**’ 0.01 ‘*’ 0.05 ‘.’ 0.1 ‘ ’ 1 \| \| \| \| \| \| --- \| --- \| --- \| --- \| --- \| \|  \|  \|  \|  \|  \| \| Residual standard error: 0.01227 on 82 degrees of freedom \| \| \| \| \| \| Multiple R-squared: 0.6575, Adjusted R-squared: 0.5907 \| \| \| \| \| \| F-statistic: 9.84 on 16 and 82 DF, *p*-value: 3.549e-13 \| \| \| \| \| | | | | | |

We generate $GR{6096}_{new}$ by calculating:

$$GR{6096}_{new}=\hat{\alpha}+0*MALARIA+\hat{\delta}_{1}OPEN+ \hat{\delta}_{2}FERTILITY+\hat{\delta}_{3}GDP60+\hat{\delta}_{4}HIGHER.EDU+\hat{\delta}_{5}INV.PRICE+\hat{\delta}_{6}LIFE.EXP+\hat{\delta}_{7}PRIM.EDU+\hat{\delta}_{8}POL.RIGHTS+\hat{\delta}_{9}POP+\hat{\delta}_{10}TROPICA+\hat{\delta}_{11}TRADE+\hat{\delta}_{12}BRIT.COL+\hat{\delta}_{13}SPAIN.COL+\hat{\delta}_{14}AREA.WATER+\hat{\delta}_{15}PUBLIC.INV+\hat{\epsilon}$$

where $\hat{\alpha}, \hat{\delta}_{1},\ldots, \hat{\delta}_{15}$ are the estimates of $\alpha, \delta_{1},\ldots, \delta_{15}$ in regression (5) in the article and $\hat{\epsilon}$ are the estimated residuals of regression (5).

**References**

1. Sala-i-Martin X, Doppelhofer G, Miller RI. Determinants of Long-Term Growth: A Bayesian Averaging of Classical Estimates (BACE) Approach. Am Econ Rev 2004;94: 813-835.
