# Supplementary material for: p-Curve and p-Hacking in Observational Research
Source: PLoS One. 2016 Feb 17;11(2):e0149144. doi: 10.1371/journal.pone.0149144 (PMC4757561; doi:10.1371/journal.pone.0149144)
Supplement: S3 Appendix — (DOCX) [file pone.0149144.s003.docx]

**S3 Appendix. Empirical Illustration for the Full Sample of 99 Countries**

We use the empirical illustration as outlined in the article but without sampling from the 99 countries to ensure that the right-skewed *p*-curve is not caused by sampling errors. Instead we use the full sample of 99 countries. The vibration plot (corresponding to Fig 2 in the article) for the 5005 models using all 99 countries is provided below.


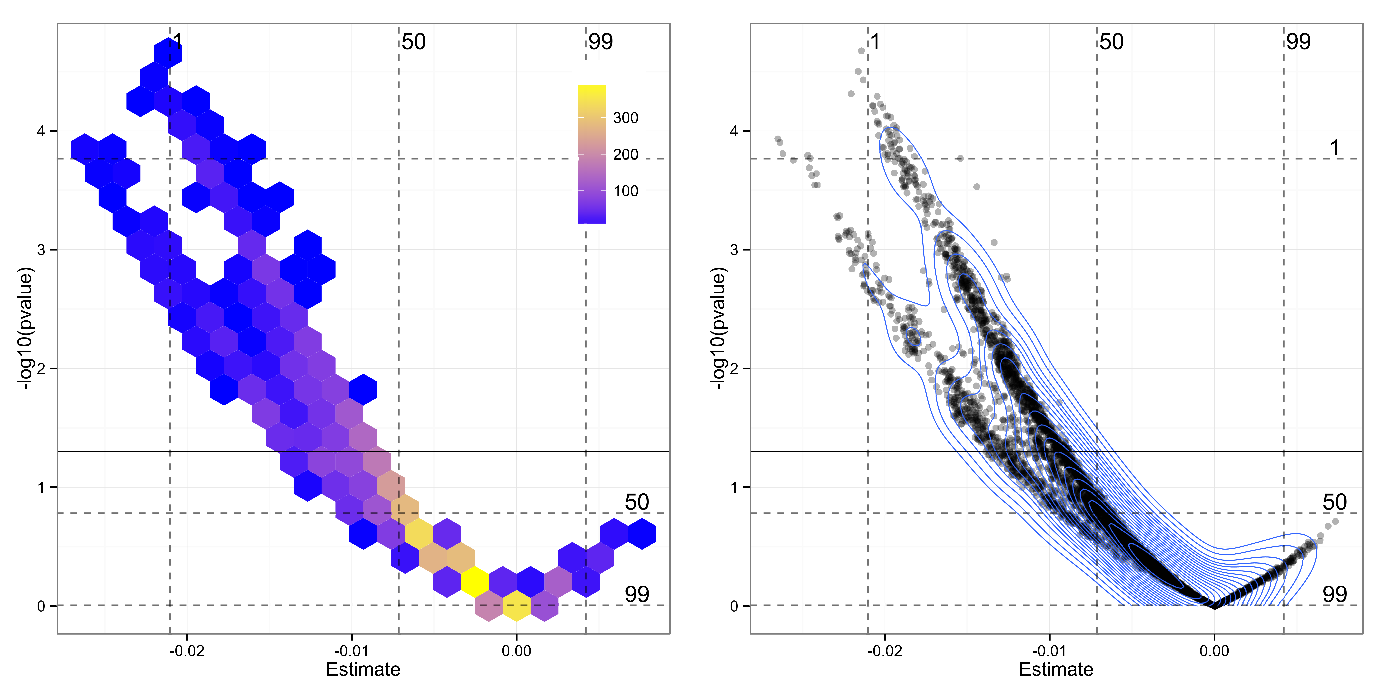


**Fig A*.* Vibration plot for the effect of malaria prevalence on economic growth for the full sample of 99 countries.** The vibration plot shows estimates of the effect of malaria prevalence in 1966 on the annualized average growth rate of real GDP per capita (1960-1996) on the x-axis. The y-axis shows transformed $p$-values of these estimates. The dashed lines represent the 1, 50, and 99 quantiles of the distribution of transformed $p$-values and of the distribution of $\beta$, respectively. The solid line represents $p=0.05$. Note that due to the transformation of $p$-values estimates above the line are statistically significant and below the line estimates are insignificant.

We search for statistically significant and negative estimates by browsing randomly through the 5005 models and stop as soon as we find a negative and statistically significant estimate. We repeat this until we obtain 100,000 negative and statistically significant estimates. The *p*-curve and the histogram of the estimates (corresponding to Fig 3 in the article) are provided below. The *p*-curve is again right-skewed, but not as smooth as with the sampling from countries. The sampling from countries ensures that there is a wider range of significance levels due to the variation of sample sizes and sampling errors.


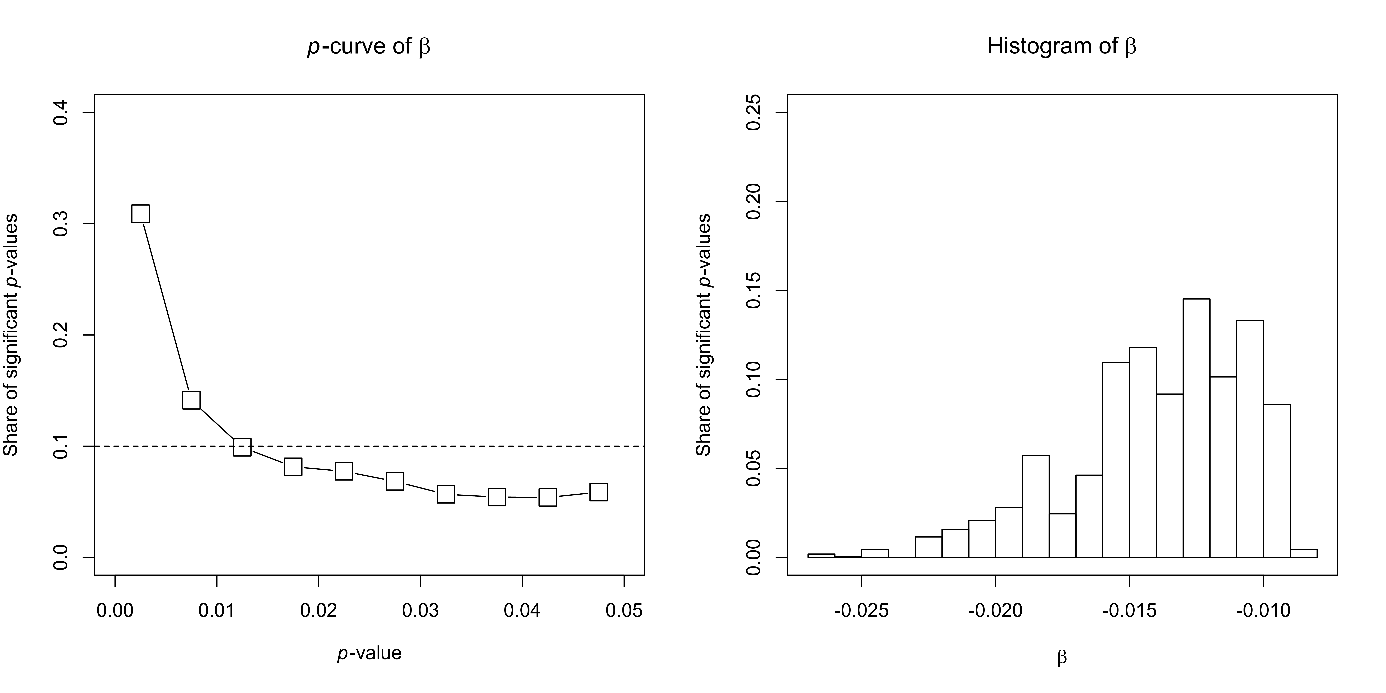


**Fig B.** $\boldsymbol{p}$**-curve and histogram of estimates for the effect of malaria prevalence on economic growth for the full sample of 99 countries.** The $p$-curve of the estimated $\beta$ of equation (6) in the article is shown in the left graph. The corresponding histogram of the estimated $\beta$ is shown in the right graph. The y-axis displays the share of significant $p$-values. The graphs are based on the $p$-values of 100,000 statistically significant and negative estimates of $\beta$.
